# Isolation of Phosphate-Solubilizing Microorganisms and the Formulation of Biofertilizer for Sustainable Processing of Phosphate Rock

**DOI:** 10.3390/life13030782

**Published:** 2023-03-14

**Authors:** Nipuni Mayadunna, Samantha C. Karunarathna, Suhail Asad, Steven L. Stephenson, Abdallah M. Elgorban, Salim Al-Rejaie, Jaturong Kumla, Neelamanie Yapa, Nakarin Suwannarach

**Affiliations:** 1 Research Center of Microbial Diversity and Sustainable Utilization, Chiang Mai University, Chiang Mai 50200, Thailand; 2 Faculty of Applied Sciences, Rajarata University of Sri Lanka, Mihintale 50300, Sri Lanka; 3 Center for Yunnan Plateau Biological Resources Protection and Utilization, College of Biological Resource and Food Engineering, Qujing Normal University, Qujing 655011, China; 4 National Institute of Fundamental Studies (NIFS), Kandy 20000, Sri Lanka; 5 School of Biology and Chemistry, Pu’er University, Pu’er 665000, China; 6 Department of Biological Sciences, University of Arkansas, Fayetteville, AR 72701, USA; 7 Department of Botany and Microbiology, College of Science, King Saud University, Riyadh 11451, Saudi Arabia; 8 Department of Pharmacology & Toxicology, College of Pharmacy, King Saud University, Riyadh 11451, Saudi Arabia; 9 Department of Biology, Faculty of Science, Chiang Mai University, Chiang Mai 50200, Thailand

**Keywords:** biofertilizer, Eppawala rock phosphate, plant bioavailability, phosphate solubilization

## Abstract

As phosphorus (P) bioavailability is limited in arable lands, chemical fertilizers are being used by farmers to increase crop production. Phosphate-solubilizing microorganisms (PSMs) increase the bioavailability of sparingly soluble inorganic and organic soil phosphorus. Therefore, the current study was an effort to evaluate the phosphate-solubilizing efficiency of PSMs using tricalcium phosphate (TCP) and Eppawala rock phosphate (ERP). The efficiency of phosphate solubilization by a series of identified isolates was compared using TCP (5 g L^−1^) and ERP (5 g L^−1^) as a P source in Pikovskava’s broth. Twelve microbial isolates that showed a higher efficiency in phosphate solubilization were selected for the production of the biofertilizer. The isolate F10 in ERP broth was characterized by the highest significant level of available phosphorus (896.98 ± 10.41) mg L^−1^, followed by F5 (*Aspergillus* sp.) in TCP broth 991.43 ± 1.37 mg L^−1^. A pot trial was carried out by using *Capsicum annuum* L. as the test plant in two soil conditions: sterilized soil and non-sterilized soil with six treatments and four replicates. The significantly highest plant height, leaf length, and width were shown by chili plants treated with the formulated biofertilizer. Therefore, the application of native PSMs appeared to be an efficient method of solubilizing sparingly soluble P compounds into plant-available forms.

## 1. Introduction

Phosphorus (P) is one of the major essential macronutrients for the biochemical, physiological, and molecular level activities of plants [1]. When considering the total soil phosphorus available to plants, only 4% exists in the orthophosphate form [2]. However, phosphates in arable soils are sufficient enough to obtain the maximum yields of crops for a long period of time [1]. Generally, phosphorus is applied to soil in the form of chemical fertilizers. As the price of conventional phosphate fertilizers is higher and hazardous to the environment, the excessive application of synthetic chemical phosphate fertilizers causes phosphate leaching, and as a result, groundwater contamination occurs [3]. Therefore, the use of rock phosphate as a phosphorus source has been considered [3]. The largest rock phosphate deposit in Sri Lanka is located at Eppawala in the Anuradhapura District. The solubility of Eppawala rock phosphate (ERP) has been known to be very low [4]. As a result, rock phosphate is chemically reacted with sulfuric acid or phosphoric acid in order to form a soluble phosphate fertilizer. This process affects the price of fertilizers and is hazardous to the environment. As an alternative to this situation, microorganisms capable of solubilizing rock phosphate can be used to release soluble phosphorus from ERP [5,6]. Although phosphate-solubilizing microbial consortia were formulated by the scientists, formulating a native efficient microbial consortium is necessary. 

Phosphate-solubilizing microorganisms (PSMs) have the ability to transform the insoluble P forms into soluble forms, which can function as biofertilizers by increasing the soluble P content in soil [7]. The use of P biofertilizers is an eco-friendly approach that can be used to treat the problem of infertile soil [8]. It has been reported that the inoculation of PSMs has increased the growth of plants. Phosphate-solubilizing microorganisms act as biofertilizers by transforming insoluble P forms into soluble P forms and making them available to growing plants. Phosphorous-solubilizing bacteria also help the growth of plants by performing functions such as synthesizing phytohormones, increasing trace element (Zn and Fe) availability, and increasing the efficiency of biological nitrogen fixation [9]. Studies related to the inoculation of PSM with plants have shown that this increased the P uptake and improved plant growth. Managing and adopting the microorganisms of the rhizosphere provide benefits such as stimulating root and shoot growth, improving shoot and root length, enhancing P availability to crops, improving the yield, and increasing shoot weight [10]. Phosphate-solubilizing microorganisms also affect the growth and development of plants [11]. Phosphate-solubilizing microorganisms possess functions other than solubilizing P. They function as biocontrol agents, by secreting antifungal compounds such as PAL, antibiotics, phenolics, siderophores, flavonoids, lytic enzymes, and hydrogen cyanide [2]. The most known phosphate biofertilizers are microorganisms of species of *Pseudomonas*, *Bacillus*, *Aspergillus*, and *Penicillium* [9].

Phosphate availability from rock phosphate can be enhanced by using techniques such as the affiliation of additives in rock phosphate, compression of rock phosphates with chemical fertilizers, partial acidulation of rock phosphate, and application of microorganisms [12]. Rock phosphates can be used in producing water-soluble P fertilizers as a raw material or being applied directly to the soil as a P source [13]. Insoluble rock phosphate is converted into soluble phosphate fertilizers by the activity of chemicals such as phosphoric acid or sulfuric acid [12]. 

Ca_3_(PO_4_)_2_ + 2H_2_SO_4_ + H_2_O **→** Ca(H_2_PO_4_)_2_·H_2_O + 2CaSO_4_ + 108.44 k cal. [14].

PSMs can release P by disintegrating rock phosphate and by producing organic acids and releasing protons due to the microbial metabolism (fermentation, oxidative respiration) which accounts for the P availability of rock phosphate [15]. Organic acids produced by microorganisms are responsible for the disintegration, concentration, and transportation of soil elements and the formation of soluble complexes along with the cations from minerals and rocks [16]. Additionally, PSMs can produce phosphatase enzymes which transform organic P into inorganic P through mineralization [17]. Microbial genera such as *Pseudomonas* and *Bacillus* play significant roles in releasing P from phosphates [18]. Moreover, phosphate-solubilizing fungi have the ability to enhance the bioavailability of P from rock phosphate; these include *Aspergillus* spp., *Trichoderma viride*, and *Penicillium* spp. [19]. To improve the efficiency of PSMs, they should be added along with organic manure or organic compost to provide the basic nutrient requirements for microorganisms [19]. The principal mechanisms of P solubilization carried by soil microbes are the secretion of mineral-dissolving compounds or complexing compounds (protons, hydroxyl ions, siderophores, and organic acid anions), the liberation of P during the degradation of the substrate, and the release of extracellular enzymes [20]. Phosphate-solubilizing microorganisms assemble soil P into plant-available forms by chelation, mineralization, and lowering soil pH. The mechanism of chelation involves the hydroxyl and carboxyl groups of acids. They transform insoluble forms into soluble forms by chelating the phosphate-bound cations [20].

The primary objective of the present study was to isolate and identify phosphate-solubilizing microorganisms from Sri Lankan soil, to use these to evaluate the potential of solubilizing ERP, to compare the efficiency of P solubilization of Eppawala rock phosphate and Tri-calcium phosphate by PSM, and to produce a phosphate biofertilizer that can be used in sustainable agriculture. Therefore, as Sri Lanka has a large deposit of rock phosphate in Eppawala, Anuradhapura, it can be utilized as a cost-effective phosphate fertilizer. Therefore, as a country, we could have complete material independence from other countries when producing phosphate fertilizers in the future. In addition, the growth performance of chili (*Capsicum annuum* L.) was assessed with the application of the produced biofertilizer. 

## 2. Materials and Methods

### 2.1. Study Site

The experiment was conducted in the Faculty of Applied Sciences, Rajarata University of Sri Lanka, Mihintale. The pot experiment was conducted under natural light conditions. The study area used receives an annual rainfall of 1000–1500 mm and the temperature varied between 28 and 32 °C throughout the year.

### 2.2. Sample Collection

Soil samples were collected from four different undisturbed areas at Kegalle (7°13’32.30’’ N, 80°22’3.34’’ E), Kandy (7°17’29.10’’ N, 80°38’12.10’’ E), Mihintale Sanctuary (8°21’9.69’’ N, 80°30’12.39’’ E), and the Eppawala rock phosphate site (8°10’22.22’’ N, 80°24’17.24’’ E). Eppawala rock phosphate was collected from Lanka phosphate Ltd. Eppawala, Anuradhapura, Sri Lanka. The elevations of the study sites ranged from Kegalle (248 m), Kandy (465 m), Mihintale (116 m), and Eppawala (106 m) above sea level. The air temperature of the study sites varied as follows. Kegalle (19.5–27.5 °C), Kandy (19.5–27.5 °C), Mihintale (24.0–28.0 °C), and Eppawala (21.5–29.5 °C). The humidity of the study sites at the time of sample collection was Kegalle (84%), Kandy (84%), Mihintale (84%), and Eppawala (84%) [21]. From each study site, soil samples were collected from four points at a depth of 0–15 cm and mixed as one composite sample. Forest litter, grass, or any materials on the soil surface were removed before sampling. After samples were brought to the laboratory, the soil was passed through a 2 mm sieve to remove rocks and other plant materials. Then, the soil was mixed thoroughly to ensure uniformity. Samples were stored in sealed polythene bags in order to make them airtight and then stored in a refrigerator at 4 °C before use. For the determination of chemical and physical characteristics, a subsample of soil was air-dried [22]. The electrical conductivity and pH values of the collected samples were measured and recorded.

### 2.3. Isolation and Identification of Phosphate-Solubilizing Microorganisms

From the collected soil samples and Eppawala rock phosphate (ERP), 1 g was obtained from each sample and suspended in 9 mL of sterile distilled water to make a soil suspension. Following this, 10-fold serial dilutions were made for each sample up to 10^−8^. Then, 1 mL from the dilutions, 10^−1^, 10^−3^, 10^−5^, and 10^−7^ were transferred to make pour plates from NA, PDA, and Pikovskaya’s medium. The plates were incubated for 5–7 days at 32 °C [23,24,25].

Phosphate-solubilizing microorganisms were identified as they produced a halozone in Pikovskaya’s medium. The Pikovskaya’s medium contained TCP, and as the PSMs could solubilize the less-soluble forms of phosphates, they utilized the phosphates in the culture media, and as a result, a clear zone was produced around the microbial colony. Fungi grown on PDA plates were subcultured and incubated for one week at 32 °C in Pikovskaya’s medium to identify the phosphate-solubilizing fungus by forming a halozone around the colony [22].

Twelve bacterial colonies, twelve fungal colonies, and an actinomycete colony with halozones in the culture medium around the colonies were selected and subcultured on Pikovskaya’s medium for further identification.

The morphological characteristics of colonies such as shape, size, margin, chromogenesis, opacity, elevation, surface, and texture were noted for each bacterial colony. Then, biochemical tests were carried out to identify the bacterial colonies at the genus level as per Bergey’s manual of systematic bacteriology (Gram stain, KOH test, and Endospore stain). Fungal isolates were identified using the morphological characteristics of hyphae, spore-forming units, and spores [22,23].

### 2.4. Evaluation of the Phosphate-Solubilizing Capability of PSMs

The phosphate-solubilizing capability of pure bacterial and fungal cultures was measured by the solubilization index. Pure cultures were grown in spread plates on Pikovskaya’s medium and incubated for 3–5 days at 32 °C. Afterwards, the diameter of the halozone produced and the diameter of the colony were measured using a Vernier caliper and recorded [22].

Solubilization index = Colony diameter + Halozone diameter/colony diameter [22].

#### 2.4.1. Quantification of Phosphate Solubilization

To quantify phosphate solubilization, pure cultures of phosphate-solubilizing microorganisms were grown in liquid Pikovskaya’s medium [22,24,26]. Later, the quantification of tricalcium phosphate as well as rock phosphate solubilization were completed. To evaluate rock phosphate solubilization, 5 g of tricalcium phosphate (TCP)/L in Pikovskaya’s medium was replaced with 5 g of Eppawala rock phosphate/L. For TCP and ERP solubilization evaluations, 80 mL of each broth was transferred into different 100 mL conical flasks. Since triplicates were carried out for each microbial isolation, there were 150 conical flasks containing 80 mL of culture broth. The pH value of the PKV culture broths containing tricalcium phosphate and Eppawala rock phosphate were measured, respectively. Then, culture broths were autoclaved under 15 lb/in² pressure and 121 °C for 20 min [26]. After that, microbial isolates were made to 1.5 × 10^8^ CFU/mL using 0.5 McFarland standard, and 1 mL of each isolate was transferred into the TCP-containing PKV broth and ERP-containing PKV broth, respectively. Triplicates were carried out for each isolate to increase accuracy, and culture media without microbial inoculations were treated as controls [24,26]. Bacteria-inoculated culture broths were incubated for five days and fungal, actinomycetes-inoculated culture broths were incubated for one week, shaken at 100 rpm at 30 °C [26]. Thereafter, cultures were centrifuged at 5000 rpm for 15 min and the supernatant was filtered through Whatman No. 1 filter paper. The filtrate was collected, and the pH value of the medium was measured with a pH meter and this value was recorded [24,26,27].

#### 2.4.2. Determination of Available Phosphorus 

To determine the available phosphorus content in the soil samples, ERP, TCP, and supernatant of microbial cultures, the vanadate-molybdate method was used [28]. 

##### Preparation of Extracting Solution

For this part of the experiment, 0.5 M sodium bicarbonate solution was used as the extracting solution. The solution was prepared by dissolving 42 g of sodium bicarbonate in 500 mL distilled water. Then, the pH of the solution was adjusted to 8.5 and the solution was diluted up to 1 L [28,29,30,31].

##### Preparation of the Vanadate-Molybdate Reagent

For the preparation of the vanadate-molybdate reagent, two solutions were prepared: solution A and solution B. Solution A was prepared by dissolving 2.7 g of ammonium molybdate-tetrahydrate in 30 mL of heated distilled water. Solution B was prepared by dissolving 0.125 g of ammonium metavanadate in 30 mL of boiling distilled water and the solution was allowed to cool and 33 mL of conc. HCl was added. Both solutions were allowed to cool to room temperature, and solution A was poured into solution B and diluted up to 100 mL [28].

##### Preparation of Standard Phosphate Solutions

For the preparation of the standard phosphate solutions, 0.2195, 0.4390, 0.6585, 0.878, and 1.0975 g of KH_2_PO_4_ were dissolved in 500 mL distilled water to produce standard 50 ppm, 100 ppm, 150 ppm, 200 ppm, and 250 ppm solutions, respectively [28,31]. Then, 35 mL from each solution was withdrawn and transferred to a 50 mL volumetric flask, 10 mL of vanadate-molybdate reagent was added, and the final volume was made up to 50 mL. Standard curves were plotted by measuring absorbance from the colorimeter at a wavelength of 420 nm [28].

##### Preparation of Unknown Soil, ERP, and TCP Solutions

First, 2.5 g of dry soil samples, ERP, and TCP were placed into wide-mouth 100 mL capacity conical flasks. Then, 50 mL of the extracting solution was added to each flask and 1g of phosphate-free activated charcoal was added to each flask, the flasks were placed on a mechanical shaker, and the latter was left to run for 30 min. Next, the suspension was filtered, and the filtrate was collected into a clean receiving flask [31].

##### Determination of P Concentration

From the soil, ERP, TCP extracts, and microbial culture supernatants, 35 mL was transferred to a 50 mL volumetric flask, and 10 mL of vanadate-molybdate reagent was added. The final volume was made up to 50 mL. The absorbance of all colored solutions was measured by a colorimeter at a wavelength of 420 nm [28].

### 2.5. Preparation of the Biofertilizer

From all the bacterial and fungal cultures, the cultures that showed the highest concentrations of available phosphorus after inoculation were selected for producing the biofertilizer (5 bacterial cultures and 6 fungal cultures). McFarland standards were then made for each bacterial and fungal culture. For the bacterial cultures, the 0.5 McFarland standard was used, while the McFarland standard 1 was used for fungal cultures [32]. After adjusting all cultures to the standard, the cultures were mixed in a sterilized reagent bottle. Pikovskaya’s broth with ERP instead of TCP was then added to the microbial culture to produce the biofertilizer. For the 300 mL of microbial culture, 200 mL of broth was added [33,34].

### 2.6. Pot Trial for Determination of the Effect of the Biofertilizer

A pot trial was carried out for a duration of one month in the plant house at the Faculty of Applied Sciences. The environmental temperature during the time in which the study was carried out ranged from 22 to 34.3 °C. Humidity during the study time was 72%. Rainfall was 43 mm. The average period of daylight was recorded as 12 h [21]. *Capsicum annuum* variety MI-2 was selected as the model plant for the pot trial of the biofertilizer. Soil for planting the chili was collected from the faculty premises and pH, available P concentration, and total bacterial, fungal, and phosphate-solubilizing microorganism counts were determined. The pot trial was carried out in two different soil conditions: sterilized soil and non-sterilized soil. For this, the soil was sterilized in an autoclave for 15 lb/in² pressure and 121 °C temperature for 15 min.

First, dried chili fruits without the application of a fungicide were selected and seeds were obtained. The seeds were surface-disinfected by soaking in 5% NaOCl for 5 min [24]. Then, the seeds were washed thrice with distilled water [24]. Afterwards, seeds were soaked in distilled water for 10 min and blotted using tissue paper [24]. Finally, the seeds were sown in nursery trays containing sterilized soil and non-sterilized soil, separately. Nursery trays were watered twice a day.

The pots for transplanting chili plants were prepared with 1.5 kg of soil per pot. Basal nutrients (N and K) were added to the pots according to the recommendation of the Department of Agriculture, Sri Lanka, two days before transplanting (Table 1) [35]. Nutrient solutions were prepared using ammonium sulfate for nitrogen and potassium chloride for potassium [35]. After 14 days of sowing, the chili plants were transferred into pots.

#### 2.6.1. Design of the Treatments

This experiment was carried out using a completely randomized block design and four replicates. The treatment combinations are given below. The treatments used for the pot trial were T1; control (normal soil), T2; ERP (soil + ERP), T3; TCP (soil + TCP), T4; ERP + biofertilizer (soil + ERP + biofertilizer), T5; TCP + biofertilizer (soil + TCP + biofertilizer); and T6; biofertilizer (soil + biofertilizer) (Table 1) [36]. 

#### 2.6.2. Application of Biofertilizer

The application of biofertilizer was carried out by mixing 20 mL of biofertilizer with 20 mL of carrier broth per pot (Table 1).

#### 2.6.3. Determining the Effect of the Biofertilizer

After two weeks of applying the biofertilizer to the plants, soil samples were taken from each pot and the pH value and the concentration of available phosphorus in each pot were determined. Along with these measurements, the growth parameters of the plant, such as plant height, length of the leaves, and leaf width per plant, were obtained. Total bacterial, fungal, and phosphate-solubilizing microorganism counts were taken for each pot.

### 2.7. Statistical Analysis

The statistical analysis was carried out using a two-way ANOVA procedure and SPSS/Minitab statistical software. Tukey’s pairwise comparison procedures were conducted to identify the significant differences at a significance level of 0.05 among treatments. To determine the correlations, Pearson’s correlation coefficients were computed for the selected variables.

## 3. Results

### 3.1. Measured Parameters of the Collected Samples

The basic parameters of soil samples collected from different localities showed significant differences in the tested parameters. The lowest pH value of the collected samples was 5.18 ± 0.01, which was the sample of Kandy. The highest pH was from the sample of Mihintale which was 7.14 ± 0.02. The highest significant electrical conductivity was recorded from the sample of Mihintale which was 540.80 ± 0.10 µs, and the lowest significant value was reported as 37.77 ± 0.12 µs which was the soil sample from the rubber plantation of Kegalle (Table 2).

### 3.2. Isolation and Identification of PSMs

**Figure 1:**
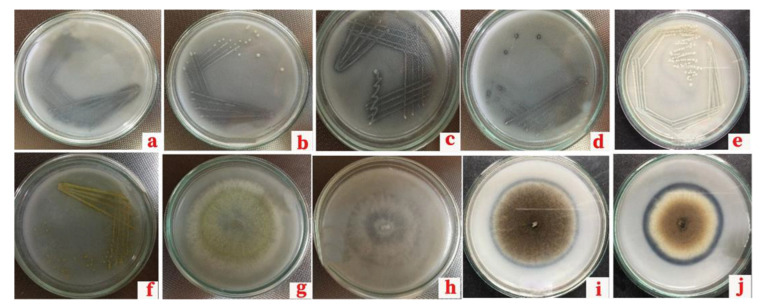
Morphological features of phosphate-solubilizing microorganisms (bacteria; (**a**) B1, (**b**) B6, (**c**) B12, (**d**) B3, (**e**) B8: actinomycete; (**f**) B13: fungi; (**g**) F11, (**h**) F4, (**i**) F8, (**j**) F10) grown on Pikovskaya’s medium.

Microorganisms producing a clear halozone on Pikovskaya’s medium were identified as phosphate-solubilizing microorganisms (Figure 1). Twelve bacterial isolates (B1–B12), eleven fungal isolates (F1–F11), and an actinomycete isolate (B13) were identified (Table 3 and Table 4). 

From the isolated bacteria, five isolates were Gram-positive and rod-shaped, four isolates were Gram-negative and rod-shaped, and three isolates were Gram-positive cocci. Only three isolates tested positive for endospore staining: B9, B11, and B12. All the isolates tested positive for the catalase test except for B1, B3, and B4, while for the oxidase test, only B5, B7, B11, and B12 tested positive. B2, B6, B7, and B9 showed positive results for the starch hydrolysis test. Only B11 tested positive for the casein hydrolysis test. B4 and B10 presented the ability of gelatin hydrolysis (Table 5). According to Bergey’s manual of systematic bacteriology, isolated bacteria belong to the genera *Bacillus*, *Pseudomonas*, and *Lactococcus*. From the lactophenol stain of the fungal isolates, they belong to *Aspergillus* sp., *Penicillium* sp., and *Trichoderma* sp. 

### 3.3. Evaluation of the Phosphate-Solubilizing Capability of Phosphate-Solubilizing Microorganisms

All the isolates showed a phosphate solubilization index (PSI) of greater than 1. The PSI of isolates ranged between 2.11 and 7.67. The highest PSI was shown by B3, while the lowest was shown by two isolates F9 and F11 (Table 6).

### 3.4. Quantification of Phosphate Solubilization

#### 3.4.1. pH Values of the Microbial-Enriched Culture Broths

**Figure 2:**
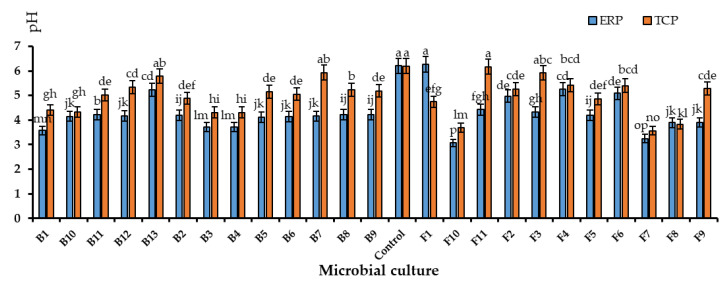
pH of microbial-inoculated ERP and TCP culture broths after incubation for 5–7 days at 100 rpm at 32 °C. Mean values that do not share the same letter are significantly different at *p* < 0.05.

The pH values of the culture broths dropped significantly compared to the controls (Figure 2). The pH value of the controls was 6.20 ± 0.01 in the TCP-enriched PK broth and ERP-enriched PK broth. The lowest pH was observed in F10 as 3.07 ± 0.05 in ERP-enriched media and 3.68 ± 0.02 in TCP-enriched media. F1 recorded the highest pH in the ERP-enriched medium as 6.27 ± 0.09. It was slightly higher than the pH of the control. The same was observed in F11, where pH increased more than the control, in the TCP-enriched broth. All the other cultures had lower pH as an indication of the production of organic acids.

#### 3.4.2. Improvement of Available Phosphorus in Microbial-Enriched Culture Broths

**Figure 3:**
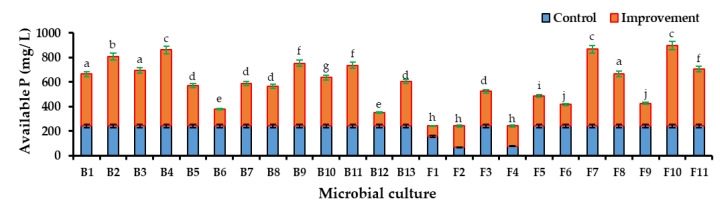
Available P concentration improvement in ERP-enriched PK broths which were inoculated with PSM cultures (B1–F11) when compared to the available P concentration in control broth. Mean values that do not share a same letter are significantly different (*p* < 0.05).

**Figure 4:**
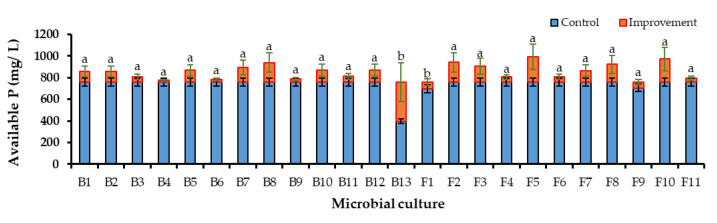
Available P concentration improvement in the TCP-enriched PK broths which were inoculated with PSM cultures (B1–F11) when compared to the available P concentration in the control broth. Mean values that do not share the same letter are significantly different (*p* < 0.05).

**Figure 5:**
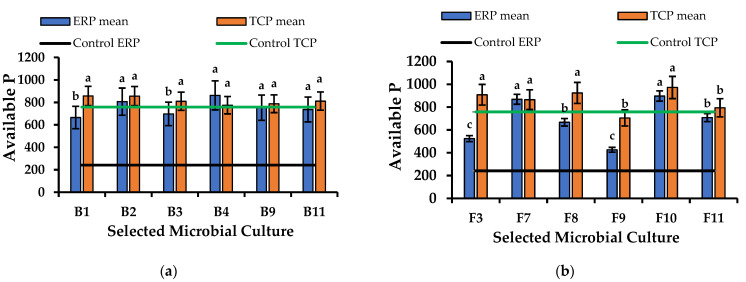
(**a**) Available P concentrations of ERP- and TCP-enriched PK broths after 5 days of inoculation with bacteria which were selected for the production of biofertilizer. (**b**) Available P concentrations of the ERP- and TCP-enriched PK broths after 7 days of inoculation with fungi which were selected for the production of biofertilizer. These had demonstrated the highest concentrations of available P when compared to the control broth. Mean values that do not share the same letter are significantly different (*p* < 0.05).

All the microbial isolates in the culture broths were shown to improve the concentration of the available P significantly. The most prominent improvement was observed in the ERP-enriched PK broths compared to the TCP-enriched PK broths (Figure 3 and Figure 4). Although the available P concentration in the control of the ERP-enriched medium was lower than the control of the TCP-enriched medium, PSMs improved the solubility of the ERP proximate to the available P level of TCP. The highest significant improvement was shown by F10 as 655.55 ± 10.41 mg/L. F7, 626.98 ± 17.88 mg/L and B4, 620.63 ± 7.94 mg/L have also shown higher improvements than other cultures. The lowest improvement was shown by B6 as 138.09 ± 17.17 mg/ L. The available P concentrations in the culture broths F1, F2, and F4 of Figure 4 and B13 of Figure 5 had reduced the available P concentration by more than that of the control. The reason for this might be the cellular uptake of dissolved P by microorganisms during cell growth.

#### 3.4.3. Selected Microorganisms for the Biofertilizer

Microbial cultures which showed the highest available P concentrations after the incubation period were selected for the production of the biofertilizer. The highest significant available P concentration was shown by B4, 620.63 ± 7.94 mg/ L in bacterial cultures (Figure 5a) and F10, 655.55 ± 10.41 mg/ L in fungal cultures (Figure 5b). Most of the selected microbial cultures for the biofertilizer improved the available phosphorus concentration of the ERP closer to the levels of the TCP.

#### 3.4.4. Correlation between Available P Concentration and pH of the ERP Broth

**Figure 6:**
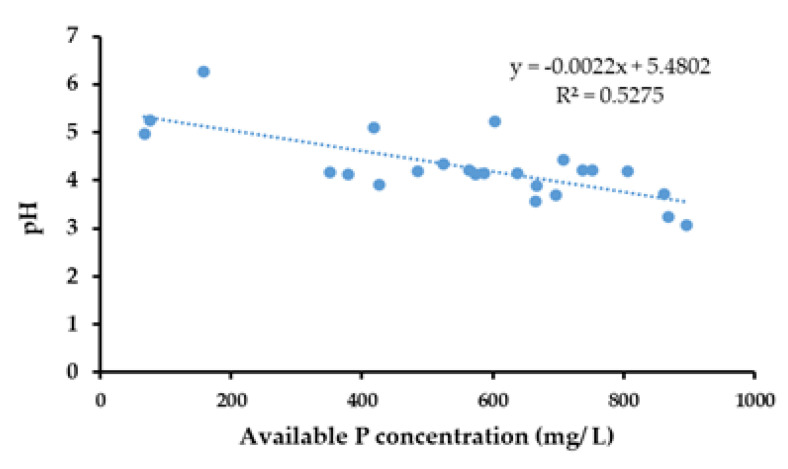
Correlation between available P and pH in the ERP-enriched PK broths inoculated with PSMs.

The results of the Pearson correlation indicated that there was a significant, very small, negative relationship between available P concentration (mg/L) and pH, (r (22) = 0.726, *p* < 0.001) (Figure 6).

### 3.5. Determining the Effect of the Biofertilizer

For the determination of the effect of the produced biofertilizer, basic soil properties and soil microbial counts were taken for the soil that had been used in the pot trials (Table 7). After the pot trials were carried out, the total microbial count was taken from each treatment to compare microbial growth..

#### 3.5.1. Electrical Conductivity

The electrical conductivity of the soil from each treatment was significantly different. The highest significant value was 650.35 ± 0.27 µS from non-sterilized treatment 3 (T3), while the lowest significant value was recorded from non-sterilized treatment 2 (T2). The values of the sterilized treatment varied in between (Table 8).

#### 3.5.2. pH

**Figure 7:**
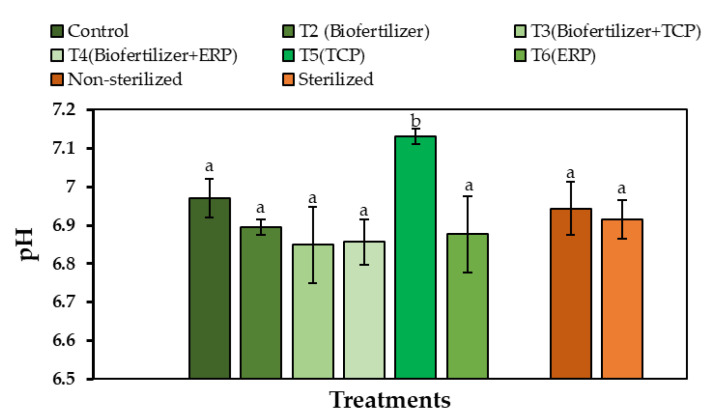
Mean values of pH of treatments. Mean values that do not share the same letter are significantly different (*p* < 0.05).

When considering the planting soil conditions, the pH values of sterilized treatments were lower than non-sterilized treatments. Treatment 5 had the highest pH value (7.13 ± 0.01) among the treatments, which was significantly higher than the control. However, in other treatments, the pH values of each treatment dropped significantly when compared to the control. The lowest pH was recorded in T3 as 6.85 ± 0.01 (Figure 7).

#### 3.5.3. Available P Concentration

**Figure 8:**
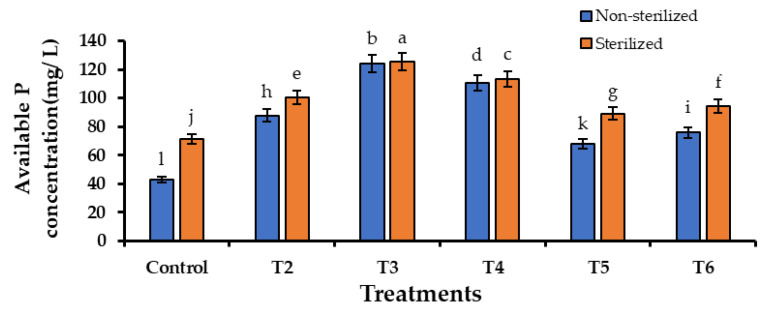
Improvement of available P compared to the control in the treatments used in the pot trial. Mean values that do not share the same letter are significantly different (*p* < 0.05).

The available P concentration for the sterilized soil conditions was significantly higher than the concentration for the non-sterilized soil conditions. In both soil conditions, available P levels in treatments were higher than in the control. The highest available P concentrations were for the biofertilizer-added treatments. The highest significant available P concentration was recorded as 124.93 ± 0.08 mg/L in T3-biofertilizer + TCP. The second highest significant value of available P was 111.88 ± 0.13 mg/L in T4-biofertilizer + ERP. The third highest value was observed in T2-biofertilizer as 94.08 ± 0.01 mg/L. The lowest significant available P value was recorded in the control treatment as 57.22 ± 0.42 mg/L. In each treatment, the available P concentration had improved (Figure 8).

### 3.6. Effect of Biofertilizer on Plant Growth

At the end of 14 days, there was a significantly higher growth of plants under sterilized soil conditions than for non-sterilized soil conditions (Table 9). Plants that were treated with the biofertilizer (T2, T3, and T4) showed the highest growth among the six treatments. When considering the height of the plant, the highest was recorded in T2 as 10.04 ± 0.53 cm. The lowest height was recorded in T6 as 8.98 ± 0.38 cm. The highest leaf length and the lowest leaf length were identified in T2 as 3.12 ± 0.09 cm, and the control as 2.8 ± 0.08 cm, respectively. After 14 days, the highest leaf width was recorded in T3 as 2.06 ± 0.15 cm. The control and T5 recorded the lowest value of leaf width as 1.78 ± 0.07 cm.

When considering the microbial population from different treatments, the highest significant count of total PSMs was recorded from the sterilized treatment 5 (T5), which was 10.95 ± 1.20 CFU/g, while the lowest significant total PSM count was recorded from non-sterilized treatment 1 (T1), which was 3.30 ± 0.58 CFU/g (Table 10).

#### 3.6.1. Correlation between Available P and pH in the Pot Trial

**Figure 9:**
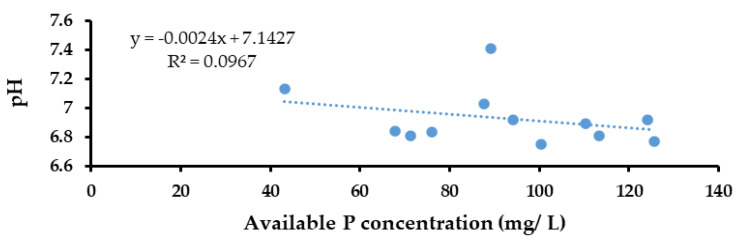
Correlation between available P and pH of soils of the different treatments used in the pot trial.

The results of the Pearson correlation indicated that there was a non-significant, very small, negative relationship between available P concentration (mg/L) and pH (r (10) = 0.311, *p* = 0.325) (Figure 9).

#### 3.6.2. Correlation between Available P and Plant Height

**Figure 10:**
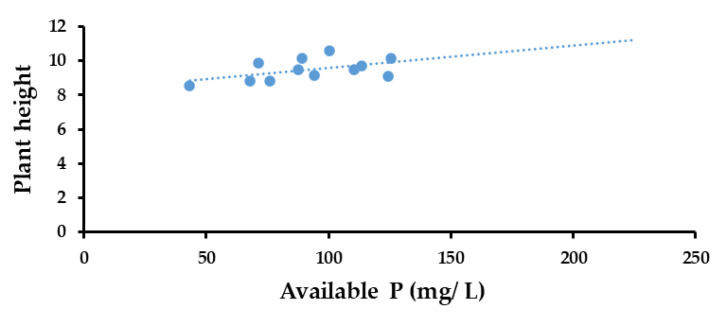
Correlation between available P and plant height for the different treatments.

The results of the Pearson correlation indicated that there was a non-significant, large, positive relationship between available P concentration (mg/L) and pH (r (10) = 0.511, *p* = 0.090) (Figure 10).

#### 3.6.3. Correlation between Total Bacterial Count and Total PSM Count in the Pot Trial

**Figure 11:**
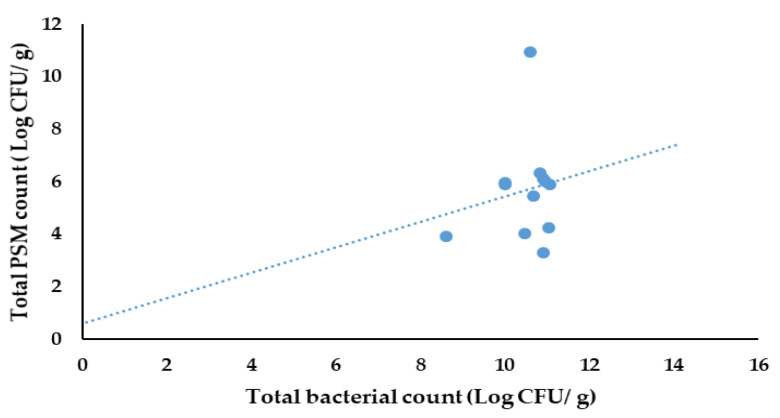
Correlation between the total bacterial count and the total PSM count for pots of different treatments.

The results of the Pearson correlation indicated that there was a non-significant, small, positive relationship between the total bacterial count and the PSM count (r (10) = 0.173, *p* = 0.591) (Figure 11).

## 4. Discussion

In the present study, microorganisms proficient in producing a halo due to the solubilization of phosphates in the nearby environment were selected as potential solubilizers of phosphates [26]. *Bacillus* sp., *Pseudomonas* sp., *Lactobacillus* sp., *Aspergillus* sp., *Penicillium* sp., and *Trichoderma* sp. were identified as potential PSMs. The bacteria were shown to produce similar results [37]. When considering fungi, Chuang et al. [38], and Onyia et al. [39] reported similar results. The activity of P solubilization was examined through the capability of PSMs by reduction of the pH due to the production of low molecular weight organic acids or alkaline phosphatases as a mechanism of P solubilization. Similar results were obtained by Chen et al. [5], Rodriguez et al. [10], and Kpomblekou et al. [40]. This finding could be supported by the results of the present study (Figure 9). According to these results, the pH values of the microbial-inoculated PK broths and soils of the biofertilizer-added treatments were significantly lower compared to the control. Nevertheless, the solubilization of phosphate is a complex process that may be influenced by many factors, including the physiological, nutritional, and growth conditions of PSMs [41]. Therefore, further studies should be conducted to understand more completely the phosphate solubilization mechanisms of different PSMs. 

Our results indicated that microbial P solubility was higher for ERP than TCP. Additionally, it was observed in our study that some microbial isolates (F1, F2, F4, and B13) reduced the available P levels in the PK broth more than was the case for the control. This could result from the cellular uptake of dissolved P by microorganisms during cell growth. The evaluation of the available P concentration using only the supernatant has the disadvantage of not considering the P utilized by the microbial cells. Therefore, an objective assessment of the ability of phosphate solubilizing in vitro is necessary [37].

Although the solubility of ERP was very low when compared to TCP, selected microorganisms for the production of biofertilizers significantly increased the solubility of ERP to near the levels of TCP. This finding suggests the potential usage of RP as an alternative to a chemical P fertilizer. 

A negative correlation was observed between pH and the available P concentration in the PK broths (Figure 7). This finding suggested that P solubilization could be expedited by acidification of the medium. A similar negative correlation between pH and the available P content was reported earlier by Illmer and Schinner [42] and Hwangbo [43]. This correlation was also observed in the pot trial between pH and the available P concentration of soil in each treatment. 

Electrical conductivity indicates the presence and absence of salts in the soil. Higher electrical conductivities in treatment soils reveal the presence of higher amounts of ions in the soil and vice versa. The study of Mylavarapu et al. [44] also showed similar results. In the present study, the electrical conductivities of soils from different treatments were significantly different from each other, and there was no observable correlation with any other factor.

Available phosphorus content in soil has been reported to improve through the inoculation of PSMs [45]. The results obtained from this study also supported this finding because, in the pot trials, plants that were treated with biofertilizer containing PSMs (T2, T3, and T4) improved the levels of available P concentration of the soil. Culture broths inoculated with microbes have also shown improved levels of available P. As per the literature, PSMs also aid in the acquirement of nutrients other than P, possibly by the production of organic acids [46]. PSMs are known to promote plant growth. The study of Amri et al. [47] showed results related to this. The results of our study were also consistent with this observation. When comparing plants grown in different treatments, a significant difference was shown by the plants which were treated with the biofertilizer (T2, T3, and T4). In addition, a positive correlation was found between the available P concentration and plant height. The non-indigenous PSM establishment of soil depends on the ability to exploit different soil substrates and the interaction with the native microorganisms associated with plants [48].

A significant difference in the growth of plants in sterilized and non-sterilized soil conditions was observed (control sterilized: 9.85 ± 0.15 cm, control non-sterilized: 8.55 ± 0.39 cm). The sterilization process changes the chemical properties of the soil and improves nutrient availability. In contrast, sterilization causes a reduction in the soil microbial population, thereby affecting the species richness and growth of successive microbial colonists [49]. This might have caused the higher growth of plants in the sterilized soil.

According to the results obtained in the present study, there was a positive correlation between the total bacterial count and the total PSM count in the soil of the pot trials. This means that native microorganisms positively support the colonization of PSMs.

## 5. Conclusions

The present study confirmed the importance of phosphate-solubilizing microorganisms on phosphorus bioavailability in soil, despite the environment-unfriendly chemical solubilization methods of phosphorus. The application of PSMs appears to be an efficient method of converting insoluble P compounds to plant-available P forms. PSMs also aid in better growth of plants, crop yield, and quality. The most efficient P solubilizers for increasing the bioavailability of P in soil are *Bacillus*, *Pseudomonas*, *Penicillium*, and *Aspergillus*. The application of PSMs is ecologically and economically beneficial. This study also demonstrated the potential of phosphate-solubilizing microorganisms as biofertilizers. According to this study, correlations between the available P concentration and pH of the surrounding environment and the available P concentration and plant growth are evident. Moreover, as PSMs have the ability to improve the solubility of ERP near the level of TCP, ERP can be used as an alternative for chemical phosphorus sources. However, technologies specific to PSMs such as isolation, growth, and processing technologies should be developed, and knowledge of PSMs should be given to farmers in the future.

## Figures and Tables

**Table 1:** The amount of basal nutrients added per pot in the different treatments.

Treatment	ERP (g)	TCP (g)	Biofertilizer (mL)	Nitrogen (g)	Potassium (g)
T1-Control	–	–	–	0.1222	0.0474
T2-ERP	0.1147	–	–	0.1222	0.0474
T3-TCP	–	0.1147	–	0.1222	0.0474
T4-ERP + Biofertilizer	0.1147	–	20.000	0.1222	0.0474
T5-TCP + Biofertilizer	–	0.1147	20.000	0.1222	0.0474
T6-Biofertilizer	–	–	20.000	0.1222	0.0474

**Table 2:** Basic chemical parameters and total microbial counts of the collected soil samples from the different localities and Eppawala rock phosphate.

Soil Sample	pH Value	Electrical Conductivity (µs)	Available Phosphorus (mg/L)	Total Bacterial Population (log CFU/g)	Total Fungal Population (log CFU/g)	Total Phosphate-Solubilizing Population (log CFU/g)
Eppawala	6.90 ± 0.01	47.68 ± 0.02	25.00 ± 0.05	10.95 ± 1.00	5.47 ± 0.50	6.23 ± 0.50
Kandy	5.18 ± 0.01	214.80 ± 0.15	39.30 ± 0.15	10.07 ± 1.00	6.60 ± 1.00	6.00 ± 0.50
Kegalle (Tea)	5.85 ± 0.02	49.49 ± 0.01	65.00 ± 0.10	10.90 ± 0.50	5.90 ± 0.50	6.14 ± 1.00
Kegalle (Rubber)	5.38 ± 0.01	37.77 ± 0.12	58.00 ± 0.05	10.84 ± 0.50	5.77 ± 1.00	6.23 ± 1.50
Mihintale	7.14 ± 0.02	540.80 ± 0.10	43.30 ± 0.15	10.14 ± 1.00	7.00 ± 3.00	6.00 ± 1.00
Eppawala Rock Phosphate	6.95 ± 0.02	72.73 ± 0.01	252.57 ± 0.31	10.69 ± 1.00	7.30 ±0.50	7.30 ± 2.50

**Table 3:** Morphological characteristics and diameter of the halozone of phosphate-solubilizing bacteria (B1–B12), and actinomycete (B13) isolated from different localities of Sri Lanka.

Isolate	Colony Diameter (mm)	Halozone Diameter (mm)	Color	Shape	Margin	Opacity	Elevation	Surface
B1	0.50 ± 0.01	2.50 ± 0.08	White	Round	Entire	Opaque	Flat	Smooth
B2	5.00 ± 0.06	6.50 ± 0.06	Cream	Round	Undulate	Opaque	Raised	Smooth
B3	0.30 ± 001	2.00 ± 0.06	White	Round	Entire	Opaque	Flat	Smooth
B4	0.50 ± 0.03	2.10 ± 0.03	White	Round	Entire	Opaque	Flat	Smooth
B5	3.00 ± 0.06	4.70 ± 0.06	White	Round	Entire	Opaque	Raised	Smooth
B6	2.00 ± 0.03	3.20 ± 0.06	White	Round	Entire	Opaque	Flat	Smooth
B7	1.50 ± 0.03	2.90 ± 0.06	White	Round	Undulate	Opaque	Raised	Smooth
B8	1.20 ± 0.04	2.90 ± 0.03	White	Round	Entire	Opaque	Flat	Smooth
B9	4.00 ± 0.06	5.80 ± 0.06	Cream	Round	Entire	Opaque	Flat	Smooth
B10	3.00 ± 0.03	4.20 ± 0.06	White	Round	Entire	Opaque	Flat	Smooth
B11	2.00 ± 0.06	3.60 ± 0.06	Cream	Round	Entire	Opaque	Raised	Smooth
B12	1.50 ± 0.03	2.70 ± 0.03	White	Round	Entire	Opaque	Flat	Smooth
B13	1.20 ± 0.03	3.10 ± 0.03	Yellow	Irregular	Lobate	Opaque	Raised	Rugose

**Table 4:** Morphological characteristics and halozone diameter of phosphate-solubilizing fungi (F1–F11) isolated from the different localities of Sri Lanka.

Isolate	Colony Color (Front Site)	Colony Color (Back Site)	Colony Diameter (mm)	Halozone Diameter (mm)
F1	Initially white, became dark green with white periphery	White	2.20 ± 0.03	3.50 ± 0.06
F2	White	White	7.00 ± 0.06	8.30 ± 0.06
F3	White	White	4.10 ± 0.06	5.80 ± 0.08
F4	White	White	8.00 ± 0.08	9.20 ± 0.06
F5	Initially white, turned black rapidly	White to pale yellow	9.00 ± 0.03	11.80 ± 0.03
F6	White	White	3.80 ± 0.06	4.90 ± 0.08
F7	Initially white, turned black rapidly	White to pale yellow	6.90 ± 0.06	8.10 ± 0.03
F8	Initially white, turned black rapidly	Pale yellow	7.60 ± 0.06	9.50 ± 0.03
F9	White	White	6.50 ± 0.03	7.20 ± 0.06
F10	Initially white, turned black rapidly	Pale yellow	7.00 ± 0.03	9.60 ± 0.06
F11	Green	White	5.20 ± 0.06	5.80 ± 0.03

**Table 5:** Results obtained from biochemical tests used for the identification of phosphate-solubilizing bacteria to the generic level that were isolated from the different localities in Sri Lanka. (+ indicates a positive test while – indicates a negative test).

Isolate	Gram Stain, Cell Shape	Endospore Stain	Catalase Test	Starch Hydrolysis Test	Casein Hydrolysis Test	Gelatin Hydrolysis Test	Oxidase Test
B1	G +, Coccus	−	−	−	−	−	−
B2	G +, Rod	−	+	+	−	−	−
B3	G +, Coccus	−	−	–	−	−	−
B4	G +, Coccus	−	−	–	−	−	−
B5	G −, Rod	−	+	–	−	−	+
B6	G −, Rod	−	+	+	−	−	−
B7	G +, Rod	−	+	+	−	−	+
B8	G −, Rod	−	+	–	−	−	−
B9	G +, Rod	+	+	+	–	−	−
B10	G −, Rod	−	+	–	–	+	−
B11	G −, Rod	+	+	–	+	–	+
B12	G +, Rod	+	+	–	–	–	+

**Table 6:** Phosphate-solubilizing index and phosphate solubilization efficiency of phosphate-solubilizing microorganisms grown on Pikovskaya’s medium, based on colony diameter and the halozone diameter of colonies.

Microbial Culture	Phosphate Solubilization Index
B1	6.00 ± 0.20
B2	2.30 ± 0.12
B3	7.67 ± 0.14
B4	5.20 ± 0.08
B5	2.57 ± 0.03
B6	2.60 ± 0.16
B7	2.93 ± 0.09
B8	3.42 ± 0.04
B9	2.45 ± 0.17
B10	2.40 ± 0.08
B11	2.80 ± 0.14
B12	2.80 ± 0.09
B13	3.58 ± 0.12
F1	2.59 ± 0.03
F2	2.18 ± 0.06
F3	2.41 ± 0.04
F4	2.15 ± 0.06
F5	2.31 ± 0.07
F6	2.29 ± 0.16
F7	2.17 ± 0.04
F8	2.25 ± 0.09
F9	2.11 ± 0.04
F10	2.37 ± 0.17
F11	2.11 ± 0.08

**Table 7:** Basic soil properties and total microbial population of the pot trial soil.

pH	E.C. (µS)	Available P Content (mg/L)	Phosphate-Solubilizing Microbial Count (CFU/g)	Total Bacterial Count (CFU/g)	Total Fungal Count (CFU/g)
7.14 ± 0.02	540.8 ± 0.31	43.303 ± 0.01	1 × 10^6^ ± 0.33	1.5 × 10^10^ ± 0.16	1 × 10^7^ ± 0.33

**Table 8:** Electrical conductivity of soil from each treatment of pot trial. Mean values that do not share the same letter are significantly different (*p* < 0.05).

Treatment	Soil Condition	Electrical Conductivity (µs)
T1-Control	Non-sterilized	541.85 ± 0.03 ^e^
T2-Biofertilizer	Non-sterilized	318.85 ± 0.10 ^l^
T3-Biofertilizer + TCP	Non-sterilized	650.35 ± 0.27 ^a^
T4-Biofertizer + ERP	Non-sterilized	452.63 ± 0 31 ^j^
T5-TCP	Non-sterilized	538.13 ± 0.08 ^f^
T6-ERP	Non-sterilized	624.58 ± 0.03 ^c^
T1-Control	Sterilized	468.13 ±0.08 ^i^
T2-Biofertilizer	Sterilized	633.10 ± 0.07 ^b^
T3-Biofertilizer + TCP	Sterilized	522.83 ± 0.05 ^g^
T4-Biofertizer + ERP	Sterilized	575.60 ± 0.11 ^d^
T5-TCP	Sterilized	444.17 ± 0.13 ^k^
T6-ERP	Sterilized	475.80 ± 0.11 ^h^

Mean values that do not share the same letter are significantly different.

**Table 9:** Effect of biofertilizer on plant height, leaf length, and leaf width of each treatment used for the pot trials.

Treatment	Plant Height (cm)	Leaf Length (cm)	Leaf Width (cm)
Sterilized soil			
T1	9.85 ± 0.15 a	3.52 ± 0.08 a	2.20 ± 0.11 a
T2	10.60 ± 0.53 a	3.70 ± 0.09 a	2.42 ± 0.08 a
T3	10.17 ± 0.47 a	3.62 ± 0.04 a	2.27 ± 0.15 a
T4	9.72 ± 0.32 a	3.65 ± 0.06 a	2.17 ± 0.15 a
T5	10.17 ± 0.52 a	3.52 ± 0.05 a	2.12 ± 0.07 a
T6	9.15 ± 0.48 a	3.62 ± 0.05 a	2.30 ± 0.11 a
Non-sterilized soil			
T1	8.55 ± 0.39 b	2.07 ± 0.39 d	1.37 ± 0.06 b
T2	9.47 ± 0.56 b	2.55 ± 0.06 bc	1.60 ± 0.04 b
T3	9.12 ± 0.60 b	2.52 ± 0.06 bc	1.85 ± 0.35 b
T4	9.47 ± 0.40 b	2.57 ± 0.02 b	1.60 ± 0.00 b
T5	8.82 ± 0.38 b	2.32 ± 0.075 bcd	1.45 ± 0.09 b
T6	8.82 ± 0.38 b	2.27 ± 0.02 cd	1.70 ± 0.08 b

Means that do not share a letter are significantly different (*p* < 0.05).

**Table 10:** Total bacterial, fungal, and PSM counts for the different soil treatments.

Treatment	Total Bacterial Count (Log CFU/g)	Total Fungal Count (Log CFU/g)	Total PSMs (Log CFU/g)
Sterilized soil			
T1	8.60 ± 0.58	3.00 ± 1.20	3.90 ± 0.33
T2	10.90 ± 1.45	5.47 ± 2.33	6.11 ± 0.58
T3	10.95 ± 0.88	5.00 ± 3.05	6.00 ± 1.85
T4	10.84 ± 3.48	5.00 ± 1.52	6.34 ± 2.18
T5	10.60 ± 1.73	3.00 ± 1.15	10.95 ± 1.20
T6	10.69 ± 2.51	3.30 ± 0.58	5.47 ± 0.58
Non-sterilized soil			
T1	10.90 ± 2.33	6.07 ± 1.45	3.30 ± 0.58
T2	11.04 ± 1.45	3.77 ± 0.58	4.25 ± 0.33
T3	11.07 ± 1.20	5.30 ± 1.52	5.90 ± 1.52
T4	10.00 ± 1.52	3.30 ± 0.58	5.95 ± 2.02
T5	10.00 ± 4.70	5.47 ± 0.88	5.90 ± 2.66
T6	10.47 ± 2.64	3.69 ± 1.20	4.04 ± 0.58

## Data Availability

Not applicable.
